# Association between serum *Chlamydia trachomatis* antibody levels and infertility among reproductive-aged women in the U.S.

**DOI:** 10.3389/fpubh.2023.1117245

**Published:** 2023-04-05

**Authors:** Peiyi Li, Zhiyun Chen

**Affiliations:** The Assisted Reproduction Center, Huizhou Central People's Hospital, Huizhou, Guangdong, China

**Keywords:** *Chlamydia trachomatis*, infertility, NHANES, Pgp3, association

## Abstract

**Introduction:**

*Chlamydia trachomatis* infection, the most prevalent sexually transmitted bacterial infection worldwide, is a significant cause of infertility. Many countries have introduced the widespread use of serologic assays for IgG seropositivity to chlamydial plasmid gene product 3 (Pgp3). However, data on the association between the level of Pgp3-IgG in the multiplex bead array assay (Pgp3AbMBA) and female infertility are still scarce.

**Methods:**

This cross-sectional analysis included 1,425 women from the National Health and Nutrition Examination Survey (NHANES) from 2013 to 2016.

**Results:**

In the fully adjusted logistic regression model, each standard deviation increments of Pgp3AbMBA (SD = 17,079.63) led to a 28% increase in the risk of infertility. The relationship remained consistent in women who had been pregnant and women who gave birth. Smooth curve fitting revealed that the association was linear across the entire range of Pgp3AbMBA. Subgroup analysis suggested that the association was significantly stronger in women who had ever used marijuana and lived in poverty.

**Conclusions:**

This study revealed a linear and independent association between the level of Pgp3AbMBA and self-reported infertility in U.S. women. Furthermore, we found that women who had ever used marijuana and lived in poverty were at the highest risk of infertility upon chlamydial infection.

## Introduction

Infertility is a serious global medical concern. Over 186 million people worldwide have infertility problems, affecting 8–12% of reproductive-aged couples (1). Infertile couples have high economic costs associated with treatments, such as intrauterine insemination and *in vitro* fertilization. Female infertility has a complex, multifactorial set of causes, including tubal pathology (2), ovulation dysfunction (3) and unexplained causes. Furthermore, an important risk factor for infertility is exposure to pathogenic microorganisms such as *Chlamydia trachomatis* and *Neisseria gonorrhoeae* (4).

*C. trachomatis* is the most prevalent sexually transmitted bacterial infection in the United States and worldwide (5) and is a significant cause of infertility. From 2013 to 2016, ~30% of U.S. women showed serological evidence of current or past infection with *C. trachomatis* (6). In 2020, 1.6 million cases of chlamydial infection were reported by the Centers for Disease Control and Prevention (CDC) (7). *C. trachomatis*-induced ascending uterine infections may result in adverse reproductive complications, such as salpingitis, pelvic inflammatory disease, tubal scarring, ectopic pregnancy and infertility (8–11). Moreover, ~50–70% of infected women are asymptomatic and undiagnosed (12–14). Untreated *C. trachomatis* infections in the genital tract may impair immune function (15–17), which was related to biological processes after semen deposition, including sperm capacitation, fertilization, embryo implantation, embryogenesis and maintenance of pregnancy.

Over recent years, the role of chlamydial infection in promoting the development of infertility has been part of an ongoing scientific debate (8, 18). A large retrospective cohort study from Denmark reported that the risk of infertility was 37% higher in women with positive *C. trachomatis* infection tests than in women with only negative tests (19). Another cross-sectional study revealed that *C. trachomatis* was one of the prevalent pathogens in patients with primary infertility (20). In addition, a report from Dutch showed that higher risk for tubal factor infertility in chlamydia-positive women (21). However, coinfections with other sexually transmitted infection (STI) are common (22); such as bacterial vaginosis in chlamydial infection (23, 24). Whether bacterial vaginosis-associated bacteria facilitate STI ascension and pelvic inflammatory disease, or whether they are the cause of STI, is difficult to determine (16). Besides, a retrospective cohort study of women in Sweden (aged < 25 in 1985) was followed up for 15 years to investigate the association between chlamydial infection and infertility (25). The cumulative incidence of infertility was reported between 3 and 7% depending on whether a woman had ever tested for or been treated for chlamydia, which was lower than expected (26). To better design interventions and policies aimed at preventing adverse trends in reproductive health, more research is needed to verify the association and to search for high-risk subpopulations.

Many countries have introduced the widespread use of *C. trachomatis* serologic assays, especially for IgG seropositivity to chlamydial plasmid gene product 3 (Pgp3) (26–28). Pgp3 has been well-recognized as the most reliable and specific marker of a previous *C. trachomatis* infection due to its high degree of conservation among clinical strains (29). Of the methods currently utilized in Pgp3-IgG testing, the multiplex bead assay (MBA) is the most sensitive (30–32). However, most of the studies investigating the relationship between chlamydial infection and infertility were based on nucleic acid amplification test, which only reported negative or positive results. Pgp3-IgG was reported to last for more than 12 years after infection and its level may reflect cumulative exposure to chlamydia (33). The risk of infertility following chlamydial infection that is identified by the quantitative Pgp3-IgG level has been scarcely reported.

Further estimates of the risk of infertility upon chlamydial infection are essential to provide needed information for clinical counseling and chlamydia surveillance of high-risk populations. Therefore, the aim of this study was to identify the association between the level of Pgp3-IgG in the multiplex bead array assay (Pgp3AbMBA) and female infertility based on the U.S. nationally representative NHANES data from 2013 to 2016.

## Materials and methods

### Study design and population

**Figure 1:**
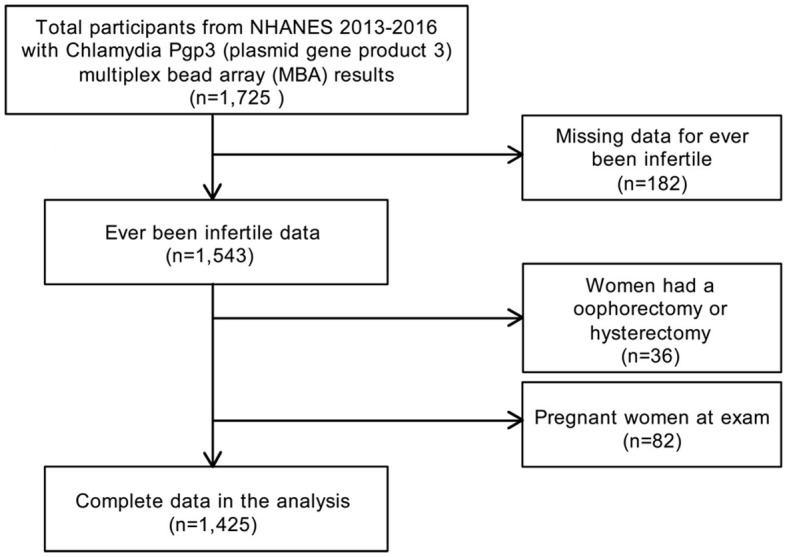
Flow chart of participant selection from the NHANES 2013–2016.

In the present study, the data were from NHANES 2013–2014 and NHANES 2015–2016, as the reproductive health questionnaires that included infertility were carried forward only for these two cycles. Using a complex multistage and stratified sampling design (34), NHANES is a biennial, nationally representative, cross-sectional survey of civilian non-institutionalized residents in the U.S. All NHANES data and more detailed information are publicly available on the CDC website (https://www.cdc.gov/nchs/nhanes/). A total of 1,726 women, aged 18–39 years, with chlamydia Pgp3AbMBA results were enrolled for NHANES 2013–2016. Participants with missing infertility data (*n* = 182), pregnant women (*n* = 82) and women with a history of oophorectomy or hysterectomy (*n* = 36) were excluded. The final analytic sample comprised 1,425 subjects (Figure 1). The NHANES study protocol was reviewed and approved by the NCHS Ethics Review Board (35). NHANES was approved by the CDC Institutional Review Board, and no written consent was required, as we conducted secondary analysis using NHANES data. Ethical approval was granted by the Ethics Committee of Huizhou Central People's Hospital (Date.20210801/No. KYLL202108). Methods adhered to the Strengthening the Reporting of Observational Studies in Epidemiology (STROBE) guidelines for cross-sectional studies (36).

### Pgp3AbMBA

The Pgp3AbMBA assay was performed by the Laboratory Reference and Research Branch in the Division of Sexual Transmitted Diseases Prevention at the CDC. After assays were verified in-house, the serum specimens were analyzed by the MAGPIX Luminex MBA system, as described previously in the literature (31, 37, 38). Briefly, beads coupled to Pgp3 antigen were incubated with 1:400 diluted serum for 1.5 h. After washing in PBST (Phosphate Buffered Saline + 0.05% Tween 20), the beads were incubated with 50 ng biotinylated mouse anti-human IgG/Fc-specific antibody (Southern Biotech, Birmingham, Alabama) and 20 ng biotinylated mouse anti-human IgG4 (Southern Biotech, Birmingham, Alabama) for 45 min. After washing in PBST, the beads were incubated with 250 ng phycoerythrin-labeled streptavidin for 30 min, washed in PBST and incubated in Buffer A (1 × PBS, 0.5% bovine serum albumin, 0.05% Tween-20 and 0.02% sodium azide) for 30 min. After washing in PBST once, the beads were suspended in 100 μl of 1 × PBS and analyzed by optical density at 511 nm. Pgp3AbMBA assays were performed only once. In the case of unreadable data or instrument error occurring the first time, the sample was rerun in a second run, and the second reading was used as the result. The results of the Pgp3AbMBA assay are expressed as median fluorescence intensity (MFI) units. Positive and negative cut-offs for the Pgp3AbMBA assays were established by testing a receiver operating characteristic panel on Pgp3-coupled beads. The MFI units above 551 were judged to be positive. Values equal to or below the cut-off were negative.

### Infertility

The outcome variable was self-reported infertility from the reproduction health questionnaires. The question was “Have you ever attempted to become pregnant over a period of at least a year without becoming pregnant?” Participants who answered “yes” were considered infertile. If the answer was “no”, then they were categorized as fertile. Otherwise, the data were regarded as missing.

### Covariates

A number of sociodemographic, lifestyle behaviors and reproductive-related variables were assessed as potential covariates. Sociodemographic variables included age, race/ethnicity (non-Hispanic White, non-Hispanic Black, Mexican American, or other Hispanic), educational levels (high school or less, some college or AA degree, or college graduate or above), poverty-income ratio (PIR) and marital status (married, never married, living with partner or divorced/separated). A PIR is calculated by dividing family income (or individual income) by poverty guidelines for the survey year. The variables of lifestyle behaviors included smoking status (current, former, or never), alcohol drinking status (had at least 12 alcohol drinks in 1 year), physical activity (moderate or vigorous work activity), energy intake (kcal per day) and drug use (ever used marijuana/hashish, ever used cocaine/heroin/methamphetamine). Anthropometric variables included body mass index (BMI) and waist circumference. Reproductive variables were based on women's self-report questionnaires. Reproductive factors included age when first menstrual period occurred, had ever taken birth control pills, had ever been pregnant, had ever gave live birth, age at first live birth, age at last live birth, ever treated for a pelvic infection (yes or no), recent gonorrhea (in the past 12 months, has a doctor or other health care professional told you that you had gonorrhea) and recent chlamydia (in the past 12 months, has a doctor or other health care professional told you that you had chlamydia).

### Statistical analysis

In the current cross-sectional analysis, Pgp3AbMBA was severely skewed toward the right. Thus, the MFI value of Pgp3AbMBA were log 10-transformed before analysis. The MFI value of Pgp3AbMBA were categorized into quintiles according to the weighted distributions of the study population. Continuous variables were tested for normal distribution using the Anderson-Darling normality test. Continuous variables are expressed as the mean ± SD, and categorical variables are expressed as percentages. We conducted weighted chi-square tests for categorical variables and weighted regression models for continuous variables. Bonferroni correction was applied to all multiple comparisons. The association of variables and self-reported infertility was first evaluated using univariate logistic regression analysis. Covariates were included as potential confounders if: (1) they changed the estimate of Pgp3AbMBA on self-reported infertility by more than 10%, (2) were known or suspected risk factors for infertility, or (3) were statistically significant in univariate analysis. We then built the logistic regression models to investigate the relationship between Pgp3AbMBA and infertility, both unadjusted and adjusted for covariates. The adjusted odds ratios (ORs) and their 95% confidence intervals (95% CIs) were calculated to evaluate the degree of association. In addition, we evaluated the linearity of the association between Pgp3AbMBA and infertility using a fully adjusted generalized additive model with a spline function. Subsequently, according to the covariates in the full model, we performed several secondary analyses, including interaction and stratified analysis. Missing values for covariates were treated as dummy variables in the models, including BMI, PIR, male sex partners, and age at first sex, and the missing ratios were 0.70, 5.82, 0.70, and 8.21%, respectively. Missing values for categorized covariates were included as an additional category. The *P*-values for interactions were tested by the likelihood-ratio test. The data were analyzed using R (http://www.R-project.org) and EmpowerStats software (X&Y Solutions). All analyses took sampling weights into account (39). A combined 4-year weight was calculated by dividing the MEC exam weight (wtmec2yr) by two. *P* < 0.05 indicated statistical significance.

## Results

### Characteristics of subjects

In total, 1,425 women were included in the study (Figure 1). When dividing Pgp3AbMBA into quintiles, a higher Pgp3AbMBA level was associated with older age, non-Hispanic black ethnicity, lower education level, unmarried status, lower PIR, higher BMI and larger waist size. In addition, higher levels of smoking, alcohol use, vigorous work activities, marijuana/hashish use, and cocaine/heroin/methamphetamine use were in quintile 5 of Pgp3AbMBA. Regarding fertility-related conditions, women with higher serology results had a lower occurrence of pregnancy and childbirth, were younger at first and last live birth, had a higher frequency of pelvic infection, had more recent history of gonorrhea prevalence, had a more recent history of chlamydia prevalence, were younger when they first had sex, had more male sex partners and engaged in less unprotected sex in the last year (Table 1). A total of 54.62% of the subjects in this study were pluriparous, and 59.09% had been pregnant at some point (Table 1).

**Table 1:** Baseline characteristics of study participants by quintiles of Pgp3AbMBA.

**Variables**	**Total**	**Quintiles of Pgp3AbMBA**					***P-*value**
		**Q1**	**Q2**	**Q3**	**Q4**	**Q5**	
**Sociodemographic variables**							
Age (years)	28.33 ± 6.25	27.37 ± 6.19	28.15 ± 6.30	28.15 ± 6.28	29.41 ± 5.99	28.65 ± 6.30	0.0030
Race/ethnicity (%)							
Mexican American	13.10	10.45	11.07	16.58	14.82	13.11	< 0.0001
Other Hispanic	18.17	21.15	16.90	18.70	15.68	18.96	
Non-Hispanic White	57.99	63.45	68.09	59.27	58.05	33.94	
Non-Hispanic Black	10.74	4.95	3.94	5.46	11.45	33.99	
Education (%)							
High school or less	26.00	20.57	21.45	18.74	34.34	38.44	< 0.0001
Some college or AA degree	36.45	44.02	32.17	30.33	35.19	43.14	
College graduate or above	29.37	25.18	37.93	40.12	25.50	12.45	
Marital status (%)							
Married	39.20	44.48	43.58	43.99	35.65	24.44	< 0.0001
Never married	32.67	32.29	34.27	24.27	27.91	46.75	
Living with partner	12.99	7.53	7.65	17.75	20.54	12.92	
Divorced/separated	7.00	5.75	6.06	3.18	10.93	9.92	
Poverty-income ratio (PIR)	2.56 ± 1.60	2.64 ± 1.61	2.75 ± 1.61	2.92 ± 1.60	2.47 ± 1.56	1.83 ± 1.35	< 0.0001
**Anthropometric variables**							
BMI (kg/m^2^)	28.46 ± 7.84	27.69 ± 7.01	27.68 ± 7.64	28.27 ± 8.21	29.45 ± 8.54	29.61 ± 7.46	0.0033
Waist circumference (cm)	93.29 ± 17.49	92.05 ± 16.24	91.67 ± 16.43	93.19 ± 18.66	94.54 ± 18.51	95.92 ± 17.35	0.0253
**Lifestyle variables**							
Smoke status (%)							
Current	44.86	38.14	35.66	48.50	49.03	50.97	0.0457
Former	17.01	29.38	12.92	15.92	15.45	16.94	
Never	38.13	32.47	51.42	35.59	35.51	32.09	
Had at least 12 alcohol drinks/1 year—yes (%)	70.06	63.19	72.55	69.11	71.82	73.46	0.0596
Vigorous work activity-yes (%)	16.53	16.49	11.49	12.86	23.74	20.21	0.0020
Moderate work activity-yes (%)	44.04	46.32	43.63	41.20	45.45	43.74	0.7794
Energy intake (kcal/d)	1,963.24 ± 769.62	1,901.87 ± 773.72	1,978.09 ± 785.36	1,971.64 ± 778.41	1,964.11 ± 729.36	2,002.19 ± 772.31	0.6445
Ever used marijuana/hashish-yes (%)	54.06	40.61	52.35	51.59	59.15	69.62	< 0.0001
Ever used cocaine/heroin/methamphetamine-yes (%)	11.58	8.09	6.73	12.88	13.93	18.92	< 0.0001
**Reproductive factors**							
Age when first menstrual period	12.62 ± 1.70	12.62 ± 1.52	12.69 ± 1.71	12.68 ± 1.76	12.55 ± 1.74	12.50 ± 1.76	0.6520
Had ever taken birth control pills -yes (%)	70.15	69.23	70.91	69.12	71.62	69.55	0.9691
Ever pregnant (%)	59.09	50.46	53.67	49.84	32.50	25.80	< 0.0001
Had ever gave live birth (%)	54.62	53.51	57.14	53.68	38.38	33.42	< 0.0001
Age at first live birth	22.28 ± 4.82	23.57 ± 4.36	23.28 ± 4.44	24.14 ± 4.89	21.28 ± 4.69	20.00 ± 4.41	< 0.0001
Age at last live birth	26.64 ± 5.16	27.23 ± 4.42	27.65 ± 4.82	27.87 ± 4.58	26.03 ± 5.47	24.77 ± 5.52	< 0.0001
Ever treated for a pelvic infection -yes (%)	3.36	0.42	1.38	2.93	5.75	7.64	< 0.0001
Recent gonorrhea (%)	0.42	0	0	0	0.7	1.4	< 0.0001
Recent chlamydia (%)	2.31	0.39	0	0.35	2.81	7.02	< 0.0001
**Sexual behaviors**							
Age at first sex (years)	17.26 ± 3.05	17.83 ± 2.96	17.60 ± 3.07	17.97 ± 3.35	16.88 ± 2.95	15.63 ± 2.06	< 0.0001
No. of lifetime male sex partner	7.53 ± 11.62	5.51 ± 8.02	5.73 ± 7.12	6.02 ± 6.99	9.29 ± 12.26	12.50 ± 19.95	< 0.0001
Sex without condom in last year							< 0.0001
Never	35.60	38.54	31.29	32.47	36.59	41.47	
< 50%	11.13	9.08	14.24	8.33	9.40	14.28	
50–100%	40.47	33.27	41.23	44.50	44.81	37.67	
Always	12.80	19.12	13.24	14.70	9.20	6.59	

95% CI of Pgp3AbMBA quintiles: Q1 (13.31, 13.86), Q2 (20.58, 21.34), Q3 (39.27, 43.44), Q4 (3,184.55, 4,459.11), and Q5 (37,911.41, 41,587.24). Mean ± SD for continuous variables. The *P*-value was calculated by a weighted linear regression model. Percentage (%) for categorical variables. The *P*-value was calculated by weighted chi-square test. All data are weighted using pooled Medical Examination Center (MEC) survey weights provided by the National Center for Health Statistics.

### Association between Pgp3AbMBA and self-reported infertility

First, univariate logistic regressions were performed to analyze associations between collected variables and infertility (Supplementary Table 1). The univariate analysis showed that age, BMI, waist circumference, ever pregnant, had given live birth, age at first live birth, age at last live birth, number of lifetime male sex partners and Pgp3AbMBA were significantly positively associated with infertility. Never married, alcohol consumption, pelvic infection, age at first sex, and dangerous sex in the last year were significantly negatively associated with infertility.

Then, we used a logistic regression model (Table 2) to explore the association between Pgp3AbMBA and self-reported infertility. In the crude model, each SD increment (SD = 17,079.63) of Pgp3AbMBA led to a 36% increase in the risk of infertility. After adjusting for age, BMI, race, marital status, PIR, education level, alcohol consumption, number of lifetime male sex partners, age at first sex, ever pregnant, pelvic infection and recent chlamydia, each SD increment of Pgp3AbMBA resulted in a 28% increase in the risk of infertility. Quintile 5 had two times the risk of quintile 1 in the fully adjusted model. We further restricted the logistic regression analysis to the women who had been pregnant and who gave birth. The linear relationship remained consistent in these subgroups. A Pgp3AbMBA cut-off value above 551 was used to categorize participants into a positive group and a negative group. However, the correlation was not significant in the positive group (Supplementary Table 2).

**Table 2:** Logistic regression of Pgp3AbMBA for the risk of infertility.

	**Crude**	**Model 1^a^**	**Model 2^b^**	Women had been pregnant^*^	Women gave birth^*^
**Variables**	***n*** = **1,425, 100%**	***n*** = **1,425, 100%**	***n*** = **1,425, 100%**	***n*** = **780, 59.09%**	***n*** = **721, 54.62%**
	**(OR, 95% CI)**	**(OR, 95% CI)**	**(OR, 95% CI)**	**(OR, 95% CI)**	**(OR, 95% CI)**
Pgp3AbMBA per 1 SD	1.36 (1.17, 1.58)	1.34 (1.15, 1.57)	1.28 (1.03, 1.58)	1.20 (1.00, 1.44)	1.28 (1.01, 1.62)
**Quintiles of Pgp3AbMBA**					
Q1 (0.95, 1.22)	Reference	Reference	Reference	Reference	Reference
Q2 (1.23, 1.41)	1.32 (0.69, 2.52)	1.23 (0.64, 2.38)	1.23 (0.63, 2.42)	1.04 (0.48, 2.28)	1.08 (0.48, 2.45)
Q3 (1.42, 1.97)	1.20 (0.61, 2.36)	1.13 (0.57, 2.25)	1.13 (0.56, 2.29)	0.81 (0.35, 1.89)	0.78 (0.32, 1.88)
Q4 (1.99, 4.19)	1.71 (0.91, 3.23)	1.30 (0.68, 2.49)	1.14 (0.58, 2.25)	1.16 (0.55, 2.45)	1.08 (0.49, 2.41)
Q5 (4.20, 4.77)	2.69 (1.47, 4.90)	2.24 (1.21, 4.14)	2.02 (1.02, 4.00)	1.62 (0.74, 3.55)	1.57 (0.68, 3.63)
*P* for trend	< 0.001	0.008	0.048	0.184	0.265

^a^Model 1 adjusted for age and BMI.

^b^Model 2 adjusted for age, BMI, race, marital status, PIR, education level, alcohol drinking, male sex partners, ever pregnant, age at first sex, pelvic infection and recent chlamydia.

^*^Covariates adjusted in the two subgroups as in Model 2, except for the stratifying variable.

### Linear association

**Figure 2:**
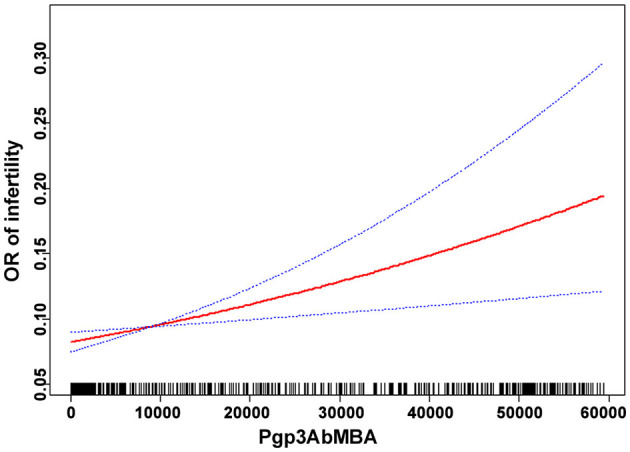
The relationship between Pgp3AbMBA and infertility by smooth curve fitting.

Smooth curve fitting was performed after adjusting for confounding factors in model 2. The results indicated that the association between Pgp3AbMBA and self-reported infertility was linear over the entire range of Pgp3AbMBA (Figure 2). This finding agreed with the stepwise increased aOR in the logistic regression analysis (Table 2).

### Subgroup analysis

**Figure 3:**
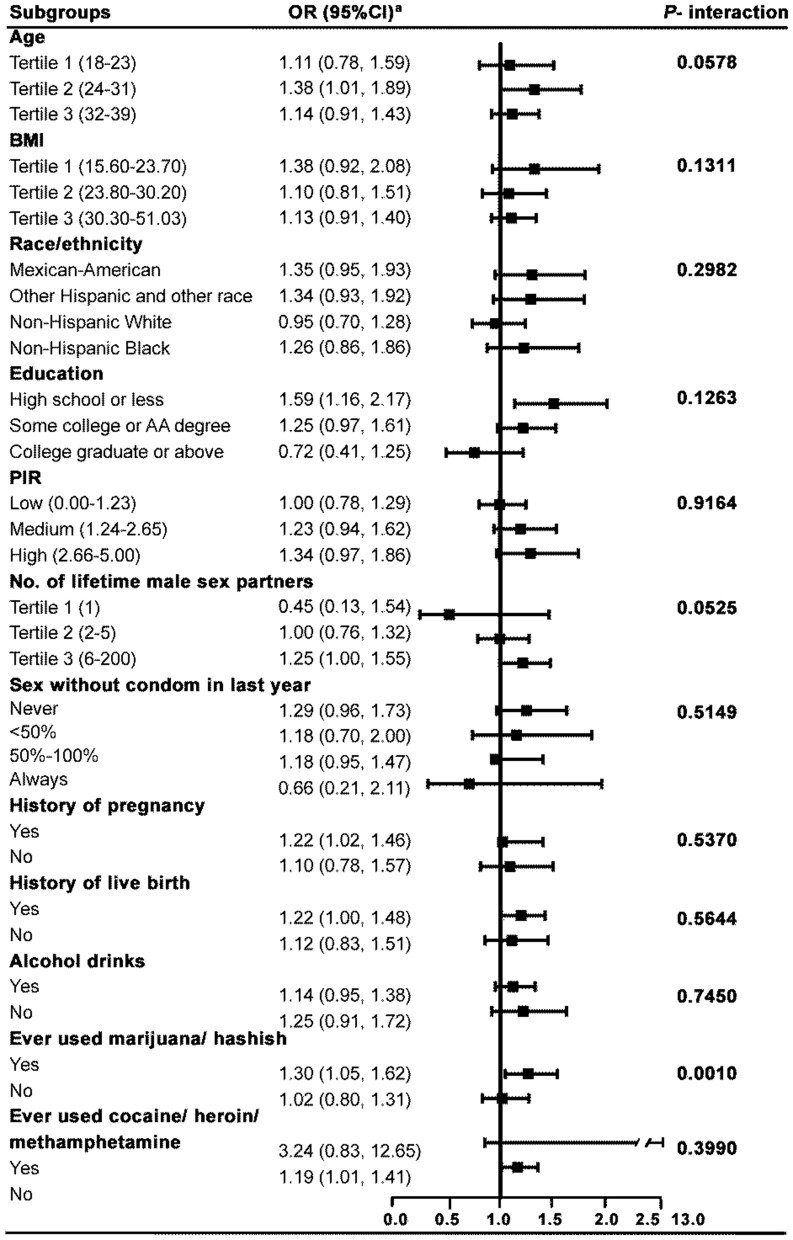
Subgroup analysis for the association between Pgp3AbMBA and infertility. ^a^Model adjusted for age, BMI, race, marital status, PIR, education level, alcohol consumption, male sex partners, ever pregnant, age at first sex, pelvic infection and recent chlamydia, except for the stratifying variable.

To determine whether the association between Pgp3AbMBA and infertility was consistent across the population groups, we conducted a subgroup analysis by numerous variables, as summarized in Figure 3. The results indicated that the association was consistent in the subgroups of age, BMI, race, education level, PIR, male sex partners, sex without condoms in the last year, ever pregnant, had ever given live birth, alcohol consumption, and ever used cocaine/heroin/methamphetamine (*P* > 0.05 for all interactions). We found that there was an interaction in the subgroup of ever used marijuana/hashish (*P* = 0.0010). The association between Pgp3AbMBA and infertility was significantly stronger in women who ever used marijuana/hashish than in those who never used marijuana/hashish (OR = 1.33; 95% CI: 1.07–1.66) (Table 3). Smooth curve fitting representing the linear association between Pgp3AbMBA and infertility in the subgroup of ever used marijuana/hashish was shown in Supplementary Figure 1.

**Table 3:** Comparison of risk of infertility following chlamydial infection in subgroups of women stratified by PIR and ever marijuana/hashish use.

**Variables**	**Crude model**		**Model 1** ^**a**^		**Model 2** ^**b**^	
	**OR (95% CI)**	***P*** **-value**	**OR (95% CI)**	***P*** **-value**	**OR (95% CI)**	***P*** **-value**
**Ever used marijuana/hashish-yes**						
PIR Q1 ( ≤ 0.95)	1.51 (1.04, 2.19)	0.0314	1.49 (0.97, 2.27)	0.0655	2.15 (1.12, 4.14)	0.0216
PIR Q2 (0.96, 1.93)	1.45 (1.05, 2.01)	0.0243	1.45 (1.05, 2.02)	0.0247	1.18 (0.74, 1.88)	0.4890
PIR Q3 (1.94, 3.27)	1.18 (0.82, 1.72)	0.3707	1.14 (0.78, 1.66)	0.5053	1.39 (0.81, 2.37)	0.2331
PIR Q4 (3.28, 5.00)	1.67 (1.12, 2.49)	0.0126	1.47 (0.96, 2.26)	0.0770	1.40 (0.82, 2.41)	0.2163
**Total**	1.44 (1.20, 1.72)	< 0.0001	1.39 (1.16, 1.68)	0.0005	1.33 (1.07, 1.66)	0.0114
**Ever used marijuana/hashish-no**						
PIR Q1 ( ≤ 0.95)	1.15 (0.78, 1.71)	0.4725	1.04 (0.68, 1.59)	0.8689	0.92 (0.49, 1.73)	0.7937
PIR Q2 (0.96, 1.93)	1.19 (0.83, 1.69)	0.3472	1.11 (0.77, 1.62)	0.5727	1.28 (0.76, 2.15)	0.3471
PIR Q3 (1.94, 3.27)	0.88 (0.54, 1.43)	0.5992	0.75 (0.44, 1.26)	0.2777	0.78 (0.36, 1.69)	0.5289
PIR Q4 (3.28, 5.00)	1.68 (1.12, 2.50)	0.0112	1.38 (0.86, 2.24)	0.1851	2.01 (0.82, 4.90)	0.1247
**Total**	1.20 (0.98, 1.46)	0.0772	1.06 (0.85, 1.30)	0.6138	1.01 (0.79, 1.30)	0.9130

^a^Model 1 adjusted for age and BMI.

^b^Model 2 adjusted for age, BMI, race, marital status, education level, alcohol drinking, male sex partners, ever pregnant, age at first sex, pelvic infection and recent chlamydia.

We found that there was an interaction between marijuana/hashish use and PIR (*P* = 0.0463, data not shown). Thus, the population was further divided into 8 groups according to the combination of marijuana/hashish use (yes or no) and PIR quartiles (Table 3). PIR values were classified by quartile: quartile 1 (Q1) ≤ 0.95; quartile 2 (Q2) 0.96–1.93; quartile 3 (Q3) 1.94–3.27; quartile 4 (Q4) 3.28–5. We found that in women who reported ever using marijuana/hashish, the risk of infertility after chlamydial infection was highest in quartile 1 of PIR (OR = 2.15; 95 CI%: 1.12–4.14).

## Discussion

In this study, we found an independent association between chlamydial infection and infertility that persisted after adjustment for confounders related to demographics, health behaviors and reproductive issues. We further revealed that the association was linear over the entire range of Pgp3AbMBA. Furthermore, the subgroup analysis indicated that people who had ever used marijuana/hashish and lived below the poverty line were more vulnerable than their counterparts. To our knowledge, this report is the first study on the independent linear association between infertility and chlamydial infection, as measured by quantitative Pgp3AbMBA levels. High risk of infertility population upon chlamydial infection was first reported in our study.

Our results agreed with a cohort study that included 857,324 UK women. Compared with women whose chlamydia testing status was negative, women whose test was positive had an 85% increased risk of infertility (40). In a study screening of chlamydia antibodies in 890 women visiting fertility clinics, chlamydia antibodies were present significantly more often in tubal factor infertility (27). However, those studies were based on qualitative data of chlamydial infection and not quantitative. In a previous relevant study utilizing NHANES data, a 2-fold higher risk of infertility was found among women with high levels of Pgp3Ab than women with negative Pgp3Ab results (41). Based on expert opinion and the assumption that the highest level of Pgp3Ab has the strongest association with infertility, they defined high-positive Pgp3Ab with two standards: MFI ≥ 50,000 or MFI ≥ 25,048. However, this cut-off was defined artificially and these grouping did not seem plausible. Besides, their analysis did not include confounding factors, which could significantly affect female infertility. In the present study, twelve confounders were adjusted in the regression model and Pgp3AbMBA was treated as a continuous variable. Our study is the first to report an independent and linear correlation between quantitative Pgp3AbMBA levels upon chlamydial infection and infertility in fully-adjusted regression model. Serological surveillance may be especially useful to estimate the cumulative risk of chlamydial infection (37, 42, 43), unlike PCR positivity, which may be more transient. In addition, our results showed that a cut-off value above 551, as suggested by the CDC as chlamydia positive, did not predict an increased risk of infertility. Smooth curve fitting revealed that the prominent association was linear across the entire range of Pgp3AbMBA, and hence, no saturation or threshold effect was present. Therefore, future studies on the relationship between chlamydial infection and infertility should be cautiously interpreted.

To check the stability of the association between Pgp3AbMBA and infertility in numerous subpopulations, we carried out subgroup analysis. The results revealed that the association remained robust in the subgroups of age, BMI, race, education level, PIR, male sex partners, sex without condoms in the last year, alcohol consumption, and ever used cocaine/heroin/methamphetamine. However, we found that some vulnerable groups were of particular interest. First, women who reported ever using marijuana/hashish were more vulnerable than their counterparts. Marijuana is the world's most common illicit drug. A retrospective study found that women who were marijuana smokers at enrolment had a higher risk of miscarriage during infertility treatment (44). Several studies have reported that anandamide and other components of the endocannabinoid system, such as cannabinoid receptors, are detectable in the fallopian tube (45, 46). Moreover, anandamide was reported mostly concentrated in the isthmus (47), where sperm capacitation and early embryogenesis occur (48). Therefore, the use of marijuana was reported to associate with female infertility, as well as abnormal embryo implantation and development (49, 50).

Second, when the study population was further classified into 8 groups based on the combination of marijuana/hashish use and PIR quartiles (Table 3), the highest risk of infertility upon chlamydial infection was seen in women who ever used marijuana/hashish and lived below poverty. PIR is calculated as the ratio of the family's income to the poverty threshold guidelines, which is specific to the appropriate year and state. A PIR under 1.0 (income less than the poverty level) represents a person living below the poverty line (51). Poverty has been demonstrated to substantially increase the risk of infertility in many aspects: limited access to health services and nutrition increases susceptibility to genital infection (52). Moreover, a recent study based on 2005 to 2018 NHANES data reported that the prevalence of past-year marijuana use significantly increased among those with income below the poverty level (53). Marijuana use and poverty promote each other and may impair health and fertility. Women living below poverty may experience increase vulnerability to the adverse effects of marijuana. In our analysis, those women with these two risk factors could be more at risk for developing a more severe genital chlamydial infection leading to infertility. Taken together, these findings indicated that marijuana, particularly coupled with substantial economic problems, may be detrimental to fertility upon chlamydial infection. Our findings emphasized that chlamydia prevention should be targeted at high-risk populations and that diagnosis must be followed by optimal treatment to reduce the burden of chlamydial infection. Overall, our study showed that marijuana users and poverty-stricken women were most likely to develop infertility following chlamydial infection. To our knowledge, such relationship has never been reported hitherto.

There were limitations to this study. First, this was a cross-sectional study, and we could not ascertain temporality or causation. Second, with a median age of ~28 years, our study cohort was relatively young; therefore, some women would not have wanted to become pregnant at this age. This could have led to an underestimation of the associations that we found. However, the association remained consistent between the two groups, which were women who had been pregnant and who gave birth. Finally, although this analysis controlled for some confounders, the findings may have been influenced by factors not accounted for, such as the reproductive factors of males, coinfection of other sexually transmitted disease and variability in Pgp3AbMBA assay.

## Conclusions

Our study revealed a positive and linear association between the Pgp3AbMBA level and self-reported infertility in U.S. women. Furthermore, we found that women who had ever used marijuana and lived in poverty were most at risk of infertility upon chlamydial infection. This indicates the need for adequate diagnosis and effective treatment for chlamydia infection, particularly for high-risk populations, and to maintain good reproductive health.

## Data availability statement

Publicly available datasets were analyzed in this study. This data can be found here: https://www.cdc.gov/nchs/nhanes/.

## Ethics statement

The studies involving human participants were reviewed and approved by Ethics Committee of Huizhou Central People's Hospital. Written informed consent for participation was not required for this study in accordance with the national legislation and the institutional requirements.

## Author contributions

PL and ZC contributed to the study conception and design. ZC participated in data analysis. PL prepared the figure and tables and wrote the manuscript text. Both authors reviewed and approved the final manuscript.
