# Supplementary material for: Association between serum Chlamydia trachomatis antibody levels and infertility among reproductive-aged women in the U.S
Source: Front Public Health. 2023 Apr 5;11:1117245. doi: 10.3389/fpubh.2023.1117245 (PMC10113615; doi:10.3389/fpubh.2023.1117245)
Supplement: Supplementary file 1 [file Table_1.DOCX]

Supplemental Table 1. univariate logistic regression analysis of potential risk factors for infertility.

| Variables | N (%) | (OR, 95% CI) | *P* value |
| --- | --- | --- | --- |
| Sociodemographic variables |  |  |  |
| Age (years) | 27.84 ± 6.58 | 1.09 (1.06, 1.13) | <0.0001 |
| Race/ethnicity (%) |  |  |  |
| Mexican-American | 281 (19.72%) | 1.0 |  |
| Other Hispanic | 398 (27.93%) | 0.59 (0.34, 1.01) | 0.0532 |
| Non-Hispanic White | 490 (34.39%) | 0.84 (0.52, 1.35) | 0.4641 |
| Non-Hispanic Black | 256 (17.96%) | 1.07 (0.63, 1.82) | 0.8022 |
| Education (%) |  |  |  |
| High school or less | 408 (28.63%) | 1.0 |  |
| Some college or AA degree | 475 (33.33%) | 1.07 (0.71, 1.61) | 0.7519 |
| College graduate or above | 331 (23.23%) | 0.63 (0.38, 1.04) | 0.0725 |
| Marital status (%) |  |  |  |
| Married | 482 (33.82%) | 1.0 |  |
| Never married | 456 (32.00%) | 0.37 (0.24, 0.59) | <0.0001 |
| Living with partner | 176 (12.35%) | 0.65 (0.37, 1.12) | 0.1218 |
| Divorced/separated | 101 (7.09%) | 0.77 (0.40, 1.47) | 0.4275 |
| Poverty-to-income ratio (PIR) | 2.24 ± 1.54 | 0.98 (0.87, 1.10) | 0.7453 |
| Anthropometric variables |  |  |  |
| BMI (kg/m^2^) | 28.56 ± 8.14 | 1.05 (1.03, 1.07) | <0.0001 |
| Waist circumference (cm) | 93.16 ± 17.95 | 1.02 (1.02, 1.03) | <0.0001 |
| Lifestyle variables |  |  |  |
| Smokers (%) |  |  |  |
| Current | 177 (49.17%) | 1.0 |  |
| Former | 58 (16.11%) | 1.08 (0.43, 2.70) | 0.8733 |
| Never | 125 (34.72%) | 1.15 (0.57, 2.32) | 0.6920 |
| Had at least 12 alcohol drinks/1 year |  |  |  |
| Yes | 910 (63.86%) | 1.0 |  |
| No | 515 (36.14%) | 0.68 (0.46, 1.00) | 0.0500 |
| Vigorous work activity-Yes (%) |  |  |  |
| Yes | 224 (15.72%) | 1.0 |  |
| No | 1200 (84.21%) | 0.95 (0.58, 1.53) | 0.8184 |
| Moderate work activity-Yes (%) |  |  |  |
| Yes | 568 (39.86%) | 1.0 |  |
| No | 857 (60.14%) | 0.92 (0.64, 1.31) | 0.6315 |
| Energy intake (kcal/d) | 1945.15 ± 780.52 | 1.00 (1.00, 1.00) | 0.0906 |
| Ever used marijuana/ hashish |  |  |  |
| Yes | 707 (50.18%) | 1.0 |  |
| No | 702 (49.82%) | 0.93 (0.65, 1.33) | 0.6800 |
| Ever used cocaine/ heroin/ methamphetamine |  |  |  |
| Yes | 139 (9.87%) | 1.0 |  |
| No | 1269 (90.13%) | 0.92 (0.52, 1.66) | 0.7905 |
| Reproductive factors |  |  |  |
| Age when first menstrual period | 12.48 ± 1.73 | 1.03 (0.93, 1.14) | 0.6322 |
| Had ever taken birth control pills (%) |  |  |  |
| Yes | 887 (62.25%) | 1.0 |  |
| No | 537 (37.68%) | 0.71 (0.48, 1.04) | 0.0754 |
| Ever pregnant (%) |  |  |  |
| Yes | 639 (44.84%) | 1.0 |  |
| No | 786 (55.16%) | 2.71 (1.80, 4.07) | <0.0001 |
| Had ever gave live birth (%) |  |  |  |
| Yes | 698 (48.98%) | 1.0 |  |
| No | 727 (51.02%) | 2.02 (1.39, 2.94) | 0.0002 |
| Age at first live birth | 21.58 ± 4.72 | 1.09 (1.04, 1.15) | 0.0005 |
| Age at last live birth | 26.46 ± 5.18 | 1.07 (1.02, 1.12) | 0.0034 |
| Ever treated for a pelvic infection -Yes (%) |  |  |  |
| Yes | 52 (3.65%) | 1.0 |  |
| No | 1363 (95.65%) | 0.29 (0.15, 0.56) | 0.0002 |
| Recent gonorrhea (%) |  |  |  |
| Yes | 6 (0.42%) | 1.0 |  |
| No | 1301 (91.30%) | 0.33 (0.04, 2.89) | 0.3187 |
| Recent chlamydia (%) |  |  |  |
| Yes | 30 (2.11%) | 1.0 |  |
| No | 1277 (89.61%) | 1.01 (0.30, 3.38) | 0.9854 |
| Sexual Behaviors |  |  |  |
| Age at first sex(years) | 17.24 ± 3.21 | 0.93 (0.87, 0.99) | 0.0203 |
| No. of lifetime male sex partners | 6.81 ± 11.11 | 1.02 (1.01, 1.03) | 0.0032 |
| Sex without condom in last year |  |  |  |
| Never | 261 (18.32%) | 1.0 |  |
| <50% | 304 (21.33%) | 1.28 (0.67, 2.45) | 0.9844 |
| 50%-100% | 143 (10.04%) | 2.23 (1.49, 3.34) | 0.5200 |
| Always | 498 (34.95%) | 0.36 (0.15, 0.86) | 0.0030 |
| Pgp3AbMBA | 5.43 ± 3.13 | 1.30 (1.15, 1.47) | <0.0001 |

Mean ± SD for continuous variables. Percentage (%) for categorical variables.
