# Supplementary material for: Association between serum Chlamydia trachomatis antibody levels and infertility among reproductive-aged women in the U.S
Source: Front Public Health. 2023 Apr 5;11:1117245. doi: 10.3389/fpubh.2023.1117245 (PMC10113615; doi:10.3389/fpubh.2023.1117245)
Supplement: Supplementary file 2 [file Table_2.DOCX]

Supplemental Table 2. Comparison of the risk of infertility for positive and negative chlamydia infection, grouped according to Pgp3AbMBA

| Variables | Crude Model | | | Model 1^a^ | | | Model 2^b^ | |
| --- | --- | --- | --- | --- | --- | --- | --- | --- |
|  | β (95% CI) | *P* value | β (95% CI) | | *P* value | β (95% CI) | | *P* value |
| Negative (≤551) | Reference |  | Reference | |  | Reference | |  |
| Positive (>551) | 2.01 (1.41, 288) | 0.0001 | 1.71 (1.18, 2.48) | | 0.0043 | 1.49 (0.96, 2.29) | | 0.0734 |

^a^ Model 1 adjusted for: age and BMI.

^b^ Model 2 adjusted for: age, BMI, race, marital status, PIR, education level, alcohol drinking, male sex partners, ever pregnant, age at first sex, pelvic infection and recent chlamydia.
